# Novel protein kinase D inhibitors cause potent arrest in prostate cancer cell growth and motility

**DOI:** 10.1186/1472-6769-10-5

**Published:** 2010-05-05

**Authors:** Courtney R LaValle, Karla Bravo-Altamirano, Karthik V Giridhar, Jun Chen, Elizabeth Sharlow, John S Lazo, Peter Wipf, Q Jane Wang

**Affiliations:** 1 Department of Pharmacology and Chemical Biology, University of Pittsburgh, Pittsburgh, Pennsylvania 15261, USA; 2 University of Pittsburgh Drug Discovery Institute, University of Pittsburgh, Pittsburgh, Pennsylvania 15261, USA; 3 Department of Chemistry, University of Pittsburgh, Pittsburgh, Pennsylvania 15261, USA

## Abstract

**Background:**

Protein kinase D (PKD) has been implicated in a wide range of cellular processes and pathological conditions including cancer. However, targeting PKD therapeutically and dissecting PKD-mediated cellular responses remains difficult due to lack of a potent and selective inhibitor. Previously, we identified a novel pan-PKD inhibitor, CID755673, with potency in the upper nanomolar range and high selectivity for PKD. In an effort to further enhance its selectivity and potency for potential *in vivo *application, small molecule analogs of CID755673 were generated by modifying both the core structure and side-chains.

**Results:**

After initial activity screening, five analogs with equal or greater potencies as CID755673 were chosen for further analysis: kb-NB142-70, kb-NB165-09, kb-NB165-31, kb-NB165-92, and kb-NB184-02. Our data showed that modifications to the aromatic core structure in particular significantly increased potency while retaining high specificity for PKD. When tested in prostate cancer cells, all compounds inhibited PMA-induced autophosphorylation of PKD1, with kb-NB142-70 being most active. Importantly, these analogs caused a dramatic arrest in cell proliferation accompanying elevated cytotoxicity when applied to prostate cancer cells. Cell migration and invasion were also inhibited by these analogs with varying potencies that correlated to their cellular activity.

**Conclusions:**

Throughout the battery of experiments, the compounds kb-NB142-70 and kb-NB165-09 emerged as the most potent and specific analogs *in vitro *and in cells. These compounds are undergoing further testing for their effectiveness as pharmacological tools for dissecting PKD function and as potential anti-cancer agents in the treatment of prostate cancer.

## Background

The PKD family is a novel family of serine/threonine kinases and diacyglycerol (DAG) receptors. Three isoforms of PKD have been identified so far: PKD1 (formerly PKCμ), PKD2, and PKD3 (PKCν) [1-4]. Originally classified as a member of the protein kinase C (PKC) family, the PKD family is now recognized as a subfamily of the calcium/calmodulin-dependent kinase superfamily, and is only distantly related to PKC in structure [5,6]. All isoforms contain a catalytic domain, a cysteine-rich DAG-binding domain (C1), and a pleckstrin homology (PH) domain that negatively regulates PKD activity [7]. DAG regulates the localization of PKD through binding to its C1 domain [4] and its activity through regulating PKC-dependent phosphorylation of PKD on serines 738 and 742 (Ser^738/742^) in the activation loop [8,9]. Rapid, early activation of PKD by PKC then leads to autophosphorylation of PKD on serine 916 (Ser^916^) and subsequent full activation of PKD [10]. Interestingly, recent evidence suggests that while Ser^742 ^transphosphorylation by PKC is required for early activation of PKD, Ser^742 ^is also a site of autophosphorylation, and that autophosphorylation at this site is required for maintaining prolonged PKD activation [11].

Since its discovery, PKD has been implicated in various cellular functions significant to tumor development including proliferation, survival, apoptosis, angiogenesis, and motility. For example, PKD activation in response to vascular endothelial-derived growth factor (VEGF) or bombesin leads to activation of extracellular signal-regulated kinase (ERK) 1/2, regulating cell proliferation in several cell types [12,13]. PKD can also be activated by oxidative stress, which modulates cell survival through the NF-κB and JNK signaling pathways [14-16]. Furthermore, PKD has been implicated in the regulation of the epithelial to mesenchymal transition in prostate cancer cells by modulation of β-catenin, and angiogenesis in vascular endothelial cells through modulating phosphorylation and nucleocytoplasmic shuttling of class IIa histone deacetylases (HDACs) [15,17]. Disruption of these fundamental pathways could potentially lead to the development, progression, and metastasis of cancer. In recent studies, PKD expression has been shown to be dysregulated in human prostate cancer tissues [18,19], implicating a role for PKD in the progression of prostate cancer. To support this, we previously reported that a knockdown of PKD3, a member of the PKD family, using siRNA caused a dramatic arrest in cell proliferation in PC3 cells [18]. Furthermore, we also found that inhibition of PKD using the novel PKD inhibitor CID755673 not only reduced proliferation in LNCaP, DU145, and PC3 cells, but also significantly slowed migration and invasion of PC3 and DU145 cells [20].

Our previous report identified CID755673 as a potent and selective PKD inhibitor with an *in vitro *IC_50 _for PKD1 of 182 nM [20]. This compound also was active in cells and inhibited multiple known biological functions of PKD. CID755673 was highly selective and did not inhibit multiple PKC isoforms tested, or CAMKIIα. This remarkable selectivity represents a significant improvement over compounds previously used to inhibit PKD, such as Gö6976, a compound known foremost for its inhibition of PKCs [21]. Despite its apparent high specificity and potent inhibition of PKD *in vitro*, its cellular activity was relatively weak. Efforts to improve the potency of this compound are imperative to ensure its effective application in cells and animals.

In this study, we present the *in vitro *and cellular activity of five novel analogs of CID755673. The analogs were synthesized with modifications to both their core structures and side chains. We show that several of these analogs exhibited increased potency toward PKD inhibition both *in vitro *and in cells. Additionally, they cause potent growth arrest, moderate cell death, and inhibition of migration and invasion in prostate cancer cells, supporting their potential for *in vivo *applications.

## Methods

### Chemicals and reagents

DMSO was purchased from Sigma. PKCα was obtained from Cell Signaling Technology and Calbiochem, PKCβI was from Cell Signaling Technology, and PKCδ was from Enzo Life Sciences. Myelin basic protein 4-14 was purchased from Sigma. CID755673 and its analogs, kb-NB142-70, kb-NB165-09, kb-NB165-31, kb-NB165-92, and kb-NB184-02, were synthesized according to standard organic synthesis procedures [22-27].

### Synthesis of CID755673

**Figure 1:**
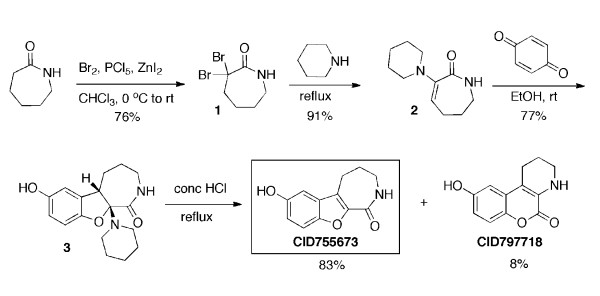
**Synthesis and chemical structures of CID755673 and CID797718**. CID755673, a compound identified and confirmed as a PKD1 inhibitor after interrogation of the PMLSC library, and CID797718, an analog of CID755673 obtained during the synthesis of the latter structure, were synthesized as described in "Methods".

CID755673 and its byproduct CID797718 were synthesized according to Fig. 1 and the following experimental protocols:

3,3-Dibromoazepan-2-one (**1**). A solution of ε-caprolactam (15.1 g, 0.133 mol) in CHCl_3 _(400 mL) was cooled to 0-5°C and PCl_5 _(55.2 g, 0.265 mol) was added over the course of 30 min followed by addition of anhydrous ZnI_2 _(1.53 g, 4.79 mmol) under N_2_. The reaction mixture was slowly allowed to reach rt as Br_2 _(42.4 g, 0.265 mol) was added dropwise over 30 min. The mixture was stirred at rt for 6 h and then poured into ice-water (300 mL). The aqueous layer was separated and extracted with CHCl_3 _(3 × 100 mL). The combined organic fractions were washed with 0.50 M aq NaHSO_3 _(3 × 200 mL) and brine (1 × 400 mL), dried (MgSO_4_), and concentrated to yield a yellow solid residue. The solid was suspended in water, filtered, and washed with water and Et_2_O to give **1 **(27.5 g, 101.5 mmol, 76% yield) as a white solid: mp 161-163°C (lit 162-164°C);[25]^1^H NMR (CDCl_3_, 600 MHz) δ 6.07 (bs, 1 H), 3.38 (dd, *J *= 10.2, 6.0 Hz, 2 H), 2.75 (t, *J *= 6.0 Hz, 2 H), 2.0 (quint., *J *= 6.0 Hz, 2 H), 1.72 (quint., *J *= 6.0 Hz, 2 H); ^13^C NMR (CDCl_3_, 150 MHz) δ 168.5, 69.5, 45.9, 42.6, 28.4, 28.2; IR (ATR, neat) 3201, 3085, 2940, 2929, 1661, 1464, 1407, 1326 cm^-1^; HRMS (ES^+^) *m/z *calcd for C_6_H_9_Br_2_NO [M+Na]^+^, 291.8949, found 291.8973.

3-Piperidin-1-yl-1,5,6,7-tetrahydroazepin-2-one (**2**). A solution of **1 **(27.0 g, 99.7 mmol) in piperidine (240 mL) was heated at reflux for 4.5 h under N_2_. The solution was allowed to reach rt and washed with 0.50 M aq NaHSO_3 _(200 mL). The aqueous phase was separated and extracted with CHCl_3 _(3 × 100 mL). The combined organic fractions were washed with brine (1 × 300 mL), dried (MgSO_4_), and concentrated to afford a yellow solid, which was suspended in water, filtered, and washed with water and Et_2_O to give **2 **(17.6 g, 90.59 mmol, 91% yield) as a white solid: mp 140-143°C (lit 139-144°C); ^1^H NMR (CDCl_3_, 600 MHz) δ 6.59 (bs, 1 H), 5.06 (t, *J *= 7.8 Hz, 1 H), 3.22 (dd, *J *= 13.2, 6.6 Hz, 2 H), 2.78 (t, *J *= 5.4 Hz, 4 H), 2.15 (dd, *J *= 14.4, 7.2 Hz, 2 H), 1.76 (quint., *J *= 6.6 Hz, 2 H), 1.65 (quint., *J *= 5.4 Hz, 4 H), 1.51 (quint., *J *= 6.0 Hz, 2 H); ^13^C NMR (CDCl_3_, 150 MHz) δ 171.2, 147.3, 105.2, 49.9 (2 C), 39.3, 30.0, 25.3 (2 C), 24.3, 21.3; IR (ATR, neat) 3193, 2950, 2923, 2935, 2855, 1655, 1605 cm^-1^; HRMS (EI^+^) *m/z *calcd for C_11_H_18_N_2_O [M]^+^, 194.1419, found 194.1422.

7-Hydroxy-10a-piperidino-2,3,4,5,5a,10a-hexahydrobenzofuro[2,3-c]azepin-1(1H)-one (**3**). A mixture of 1,4-benzoquinone (0.568 g, 5.15 mmol) and enamine **2 **(1.00 g, 5.15 mmol) in anhydrous EtOH (4 mL) was stirred for 11 h at rt. The precipitate was filtered off, washed with absolute EtOH and dried under high vacuum to give **3 **(1.12 g, 3.704 mmol, 77% yield) as a light pink solid: mp 254-255°C (lit 257-260°C);[27]^1^H NMR (DMSO-*d6*, 600 MHz) δ 8.73 (s, 1 H), 7.60 (t, *J *= 7.2 Hz, 1 H), 6.55 (d, *J *= 1.8 Hz, 1 H), 6.52 (d, *J *= 9.0 Hz, 1 H), 6.48 (dd, *J *= 9.0, 2.4 Hz, 1 H), 3.91 - 3.99 (m, 1 H), 3.28 (d, *J *= 12.6 Hz, 1 H), 2.91 - 2.98 (m, 1 H), 2.60 - 2.66 (m, 2 H), 2.39 - 2.46 (m, 2 H), 1.92 (d, *J *= 13.8 Hz, 1 H), 1.68 (quint., *J *= 4.5 Hz, 2 H), 1.32 - 1.49 (m, 7 H); ^13^C NMR (DMSO-*d6*, 150 MHz) δ 169.9, 151.3, 151.1, 130.1, 115.0, 111.1, 108.4, 107.4, 47.2, 46.9 (2 C), 38.2, 29.7, 27.1, 26.4 (2 C), 24.8; IR (ATR, neat) 3259 (br), 2949, 2849, 2824, 1649, 1472, 1351, 1208 cm^-1^; HRMS (EI^+^) *m/z *calcd for C_17_H_22_N_2_O_3 _[M]^+^, 302.1630, found 302.1625.

7-Hydroxy-2,3,4,5-tetrahydro-[1]benzoxolo[2,3-c]azepin-1-one **CID755673 **and 9-hydroxy-1,2,3,4-tetrahydrochromeno[3,4-b]pyridin-5-one **CID797718**. Adduct **3 **(2.0 g, 6.61 mmol) was suspended in conc HCl (6 mL) and the reaction mixture was heated at 100°C for 3 h under N_2_. After cooling the solution down to rt, a light amber precipitate was formed, which was washed with Et_2_O and filtered. The solid was dissolved in the minimum amount of MeOH, preadsorbed on SiO_2 _and purified by chromatography on SiO_2 _(5% MeOH in CH_2_Cl_2 _to *i*-PrOH, 100%), to yield **CID755673 **(1.19 g, 5.48 mmol, 83% yield) and **CID797718 **(0.118 g, 0.543 mmol, 8% yield). **CID755673**: mp (*i-*PrOH) 245-247°C (lit. 244-247°C);[27]^1^H NMR (DMSO-*d6*, 600 MHz) δ 9.36 (s, 1 H), 8.09 (t, *J *= 4.8 Hz, 1 H), 7.41 (d, *J *= 9.0 Hz, 1 H), 6.92 (d, *J *= 2.4 Hz, 1 H), 6.90 (dd, *J *= 9.0, 2.4 Hz, 1 H), 3.24 (dd, *J *= 9, 4.8 Hz, 2 H), 2.89 (t, *J *= 6.6 Hz, 2 H), 1.98 - 2.02 (m, 2 H); ^13^C NMR (DMSO-*d6*, 150 MHz) δ 161.9, 153.9, 148.1, 144.3, 129.6, 123.5, 116.9, 112.4, 105.1, 41.2, 26.8, 24.3; IR (ATR, neat) 3187 (br), 3059, 2921, 1680, 1579, 1472, 1435, 1339, 1166 cm^-1^; HRMS (ES^+^) *m/z *calcd for C_12_H_11_NO_3 _[M+H]^+^, 218.0817, found 218.0832; **CID797718: **mp (*i-*PrOH) 217-218°C (lit. 213-216°C);[27]^1^H NMR (DMSO-*d6*, 600 MHz) δ 9.41 (s, 1 H), 7.09 (d, *J *= 9.0 Hz, 1 H), 6.74 (d, *J *= 3.0 Hz, 1 H), 6.66 (dd, *J *= 9.0, 3.0 Hz, 1 H), 5.91 (s, 1 H), 3.22 - 3.24 (m, 2 H), 2.59 (t, *J *= 6.6 Hz, 2 H), 1.85 - 1.90 (m, 2 H); ^13^C NMR (DMSO-*d6*, 150 MHz) δ 158.1, 154.5, 140.9, 129.8, 122.9, 116.8, 114.8, 113.4, 106.4, 40.3, 21.6, 20.6; IR (ATR, neat) 3401, 3305 (br), 2937, 2879, 1662, 1583, 1449, 1342, 1219, 1184 cm^-1^; HRMS (ES^+^) *m/z *calcd for C_12_H_11_NO_3 _[M+H]^+^, 218.0817, found 218.0802.

### In Vitro Radiometric PKD or CAMK Kinase Assay

*In vitro *radiometric kinase assays were conducted as previously described [20]. Briefly, 1 μCi [γ-^32^P] ATP (PerkinElmer Life Sciences), 70 μM ATP, 50 ng purified recombinant human PKD1 (Biomol International, Plymouth Meeting, PA), PKD2 (SignalChem, Richmond, BC, Canada), or CAMKIIα (Enzo Life Sciences) or 75 ng PKD3 (Enzo Life Sciences), and 2.5 μg syntide-2 (Sigma) in 50 μl kinase buffer containing 50 mM Tris-HCl, pH 7.5, 4 mM MgCl_2_, and 10 mM β-mercaptoethanol. For the CAMK assay, 0.5 mM CaCl_2 _and 30 ng/μl calmodulin were pre-incubated for 10 min on ice, and then added to each reaction mixture. The reaction was incubated at 30°C for 10 min, and 25 μl of the reaction mixture was then spotted onto Whatman P81 filter paper (Whatman Inc., Clifton, NJ). The filter papers were washed 3 times in 0.5% phosphoric acid, air-dried, and counted using a Beckman LS6500 multipurpose scintillation counter (Beckman).

### In Vitro Radiometric PKC Kinase Assay

The PKC *in vitro *kinase assays were performed as described previously [20].

### Cell Lines and Western Blot Analysis

DU145 and PC3 cells were maintained in RPMI 1640 supplemented with 10% fetal bovine serum (FBS) and 1000 units/l penicillin, and 1 mg/ml streptomycin in 5% CO_2 _at 37°C. LNCaP cells were maintained as described previously [18]. Western blot analysis was carried out as previously reported [28]. Briefly, cells were lysed in lysis buffer containing 200 mM Tris-HCl, pH 7.4, 100 μM 4-(2-aminoethyl) benzenesulfonyl fluoride, 1 mM EGTA, and 1% Triton X-100. Protein concentration was determined using the BCA Protein Concentration Assay reagent kit (Pierce) and then equal amounts of protein were subjected to SDS-PAGE followed by electrotransfer to nitrocellulose membranes. Membranes were blocked with 5% nonfat milk in Tris-buffered saline and then probed with primary antibodies for either p-S916-PKD1 (Millipore), p-S742-PKCμ/PKD (Biosource), or GAPDH, followed by anti-mouse or anti-rabbit secondary antibodies conjugated to horseradish peroxidase (Bio-Rad). The enhanced chemiluminescence (ECL) Western blotting detection system (Amersham Biosciences) was used to facilitate detection of protein bands.

### MTT Assay

PC3 cells were seeded into 96-well plates (3000 cells/well) and allowed to attach overnight. Cells were then incubated in media containing 0.3-100 μM inhibitors for 72 h. 3-(4,5-Dimethylthiazol-2-yl)-2,5-diphenyltetrazolium bromide methyl thiazolyl tetrazolium (MTT) solution was prepared at 2 mg/ml concentration in PBS, sterilized by filtering through a 0.2 μm filter, and wrapped in foil to protect from light. 50 μl MTT solution was added to each well and incubated for 4 h at 37°C. Then, media was removed and 200 μl DMSO was added to each well. The plate was mixed for 5 min and the optical density was determined at 570 nm.

### Cell Proliferation Assay and Cell Cycle Analysis

Proliferation of PC3 cells was measured by counting the number of viable cells upon trypan blue staining as previously described [18]. Cell cycle analysis was performed as described [18]. Briefly, PC3 cells were treated with indicated compounds at 10 μM concentration for 48 h, and then fixed in 70% ice-cold ethanol overnight and labeled with propidium iodide. The labeled cells were analyzed using a FACScan Benchtop Cytometer (BD Biosciences).

### Wound Healing Assay

Wound-induced migration was measured as described previously [20]. Briefly, PC3 or DU145 cells were grown to confluence in 6-well plates. Migration was initiated by scraping the monolayer with a pipette tip, creating a "wound." The indicated concentration of compound was added to the media, and the wound was imaged immediately under an inverted phase-contrast microscope with 10× objective. After 24 h, cells were fixed in methanol and stained with 1% crystal violet, and a final image was taken. The wound gap was measured, and % wound healing was calculated. The average % wound healing was determined based on at least 9 measurements of the wound gap.

### Matrigel Invasion Assay

DU145 cells (8.0 × 10^4 ^cells/ml) in RPMI containing 0.1% fetal bovine serum (FBS) were seeded into the top chamber of BioCoat control inserts (pore size 8 μm) or BioCoat Matrigel invasion inserts with Matrigel-coated filters (BD Pharmingen). To stimulate invasion, media in the lower chamber of the insert contained 20% FBS. Inhibitors were added at 10 μM concentration to both the upper and lower chambers, and cells were incubated for 22 h. After incubation, noninvasive cells were removed using a cotton swab, and invasive cells were fixed in 100% methanol and stained with 1% crystal violet. After staining, cells were counted under a microscope (200× magnification). The percentage invasion was determined by cell counts in 5 fields of the number of cells that invaded the Matrigel matrix relative to the number of cells that migrated through the control insert.

### Statistical Analysis

Statistical analysis was completed using GraphPad Prism V software. A *p *value of < 0.05 was considered statistically significant.

## Results

### Design of CID755673 analogs

CID755673 and CID797718, a structural analog of CID755673, were synthesized by the PMLSC Chemistry Core following the scheme illustrated in Fig. 1 (see "Methods" for details of the synthesis). CID797718 is a byproduct of CID755673 synthesis, and has 10-fold less inhibitory activity toward PKD than the parental compound [20].

**Figure 2:**
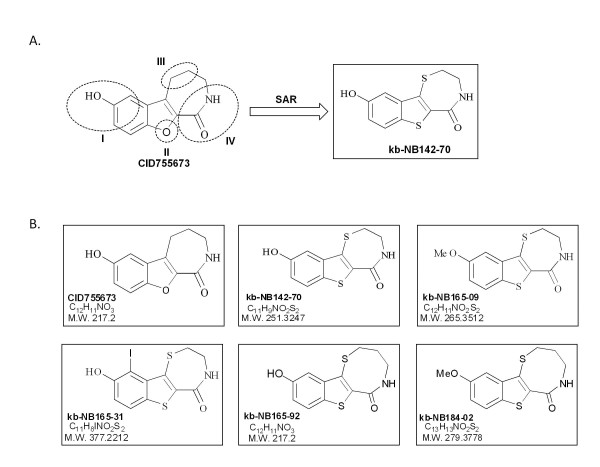
**Chemical structures and SAR of CID755673 and its analogs**. ***A***, Diagram describing the major structural zones dissected for SAR analysis. ***B***, Chemical structures of the parental compound CID755673, previously identified and confirmed as a pan-PKD inhibitor, and of five analogs of CID755673.

The design of the CID755673 analogs was based on initial structure-activity relationship (SAR) analysis described in a separate manuscript (Bravo-Altamirano K, LaValle CR, Byerly R, Giridhar KV, Chen J, Leimgruber S, Barrett R, Sharlow ER, Lazo JS, Wang QJ, Wipf P. Synthesis and Structure-Activity Relationship Evaluation of Selective Small-Molecule Inhibitors for Protein Kinase D, *manuscript submitted*). We dissected the parent compound CID755673 into 4 major structural zones in order to elucidate a fundamental SAR (Fig. 2A). In zone I, we modified the phenolic substituent as well as the α-position on the aromatic ring. In zone II, we substituted the oxygen ring atom with sulfur and nitrogen. In zone III, we altered the ring size by adding or removing methylene groups, as well as substituting the benzylic position. In zone IV, we pursued functional group interconversions as well as replacement of the amide with heterocyclic groups. Most of the zone I derivatives were considerably less active than CID755673 in the PKD screen. In particular, carbon substituents *ortho *to the phenol and *O*-benzylations were detrimental. In contrast, *ortho*-halogenation and *O*-methylation were well tolerated. Nitrogen replacements in zone II were associated with loss of activity, whereas sulfur substitution was not only tolerated well but lead often to a substantial increase in activity. Among the zone III substitutions, a thioether insertion *exo *to the five-membered heterocycle and an additional methylene group (leading to an eight-membered fused ring) were well tolerated. Finally, all zone IV substitutions were unsatisfactory, and we decided to retain the amide function of CID755673 in this position.

After initial screening and the SAR analysis on ca 50 analogs summarized above, five novel compounds with equal or greater potency for PKD were selected for further testing (Fig. 2B).

### In vitro activities of CID755673 analogs

**Figure 3:**
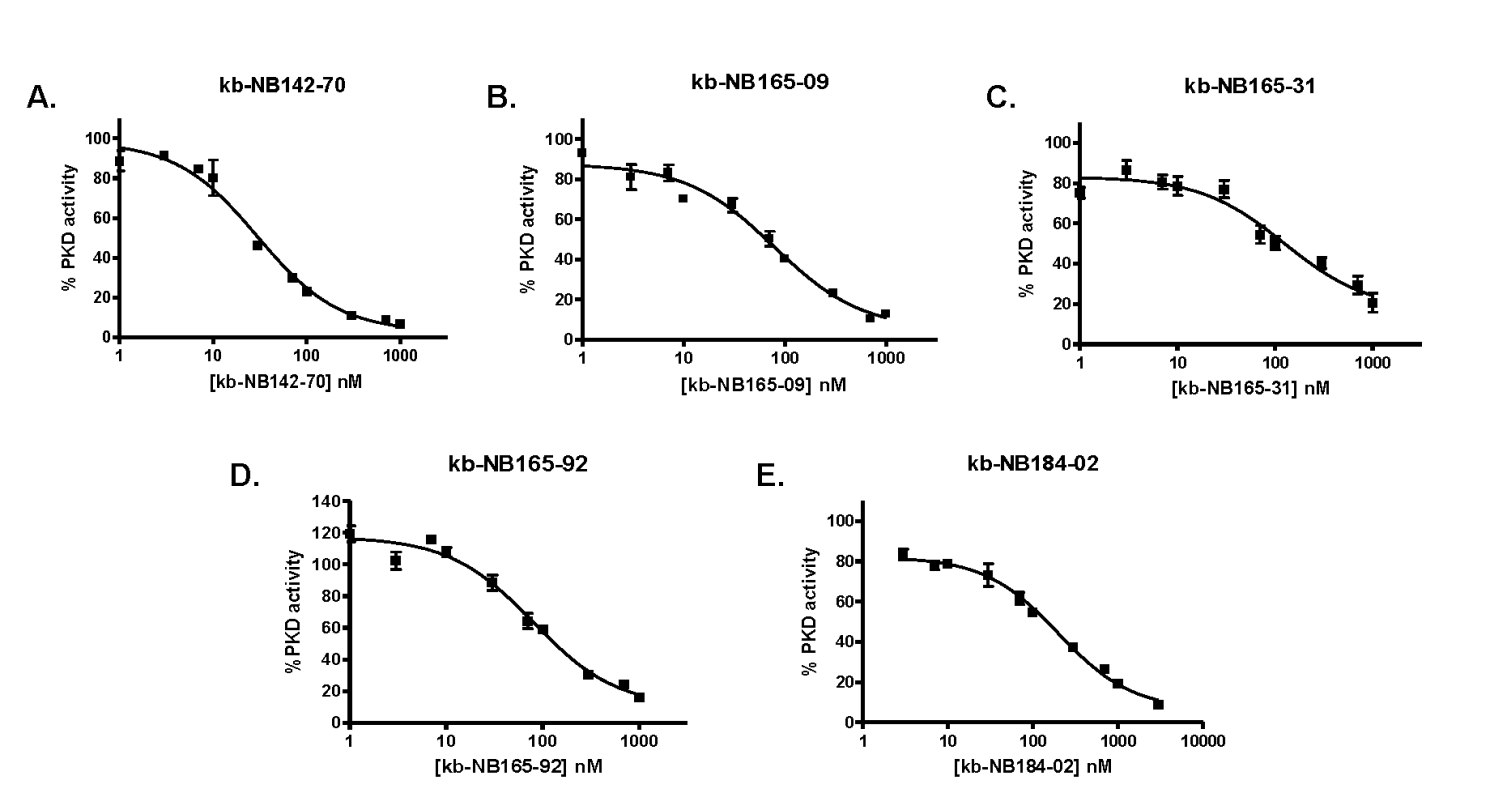
**Inhibition of PKD by CID755673 analogs *in vitro***. ***A-E***, inhibition of recombinant human PKD1 *in vitro*. PKD kinase activity was assayed by a radiometric kinase assay in the presence of increasing concentrations of the CID755673 analogs. A 10-point concentration curve was generated for each compound for IC_50 _determination. Each IC_50 _was determined as the mean ± S.E.M. of three independent experiments with triplicate determinations at each concentration in each experiment. Representative graphs are shown.

The *in vitro *inhibitory activities of the novel compounds toward PKD were determined using radiometric PKD kinase activity assays. Recombinant human PKD1, -2, or -3 was incubated with the substrate, syntide-2, in the presence of 10 different concentrations of each compound. IC_50 _values were determined for each compound by plotting percent PKD activity versus compound concentration for each point. We found that while the compounds inhibited all three PKD isoforms, their potency and selectivity varied (Fig. 3A-E and Table 1). The most potent compound was found to be kb-NB142-70, which inhibited PKD1 with an IC_50 _of 28.3 ± 2.3 nM (n = 3), showing a 7-fold greater inhibition than the parental compound (Table 1). This compound was also a robust inhibitor of PKD2 and -3, demonstrating respective IC_50_s of 58.7 ± 4.2 nM (n = 3) and 53.2 ± 3.5 nM (n = 3). Notably, kb-NB142-70 and kb-NB184-02 exhibited about 2-fold greater selectivity toward PKD1. In contrast, the compound kb-NB165-92 was more selective toward PKD3, showing approximately 2-fold greater inhibition of PKD3 (IC_50 _= 58.8 ±7.3 nM, n = 3) than PKD1 or -2 (IC_50 _= 111.2 ± 6.0 and 100.7 ±10.9, n = 3, respectively), which is unique among the compounds tested. Other compounds, namely kb-NB165-09 and kb-NB165-31 showed similar inhibition of all three isoforms. Overall, our results demonstrated that core structural modification of CID755673 substantially enhanced its potency, but had less effect on isoform selectivity.

**Table 1:** *In vitro *inhibitory activity of CID755673 and its analogs for PKD

IC_50 _(nM)			
Compound	PKD1	PKD2	PKD3
CID755673	182 ± 27 (n = 5)	280 ± 1.8 (n = 3)	227 ± 24 (n = 3)
Kb-NB142-70	28.3 ± 2.3 (n = 3)	58.7 ± 4.2 (n = 3)	53.2 ± 3.5 (n = 3)
Kb-NB165-09	82.5 ± 4.6 (n = 4)	141.6 ± 7.4 (n = 3)	98.5 ± 15.3 (n = 3)
Kb-NB165-31	114.1 ± 23.9 (n = 3)	162.9 ± 20.5 (n = 3)	91.1 ± 17.2 (n = 3)
Kb-NB165-92	111.2 ± 6 (n = 3)	100.7 ± 10.9 (n = 3)	58.8 ± 7.3 (n = 3)
Kb-NB184-02	192.8 ± 27.4 (n = 3)	463.2 ± 38.2 (n = 4)	324.7 ± 39.0 (n = 3)

IC_50 _were determined for CID755673 and its analogs against PKD1, -2, and -3 using radiometric kinase activity assays. Each IC_50 _was calculated as the mean ± S.E.M. of at least three independent experiments with triplicate determinations at each concentration in each experiment as described in "Methods." *n, number of independent experiments

### The analogs inhibit PMA-induced endogenous PKD1 activation

**Figure 4:**
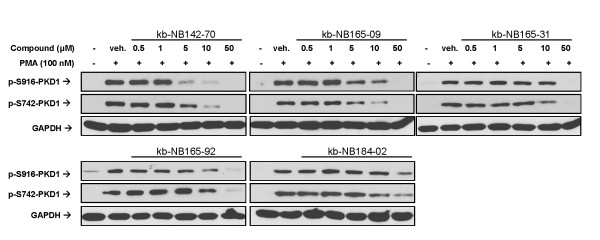
**Inhibition of PMA-induced endogenous PKD1 activation in LNCaP cells**. LNCaP cells were pretreated with indicated concentrations of the five analogs for 45 min, then stimulated with 100 nM PMA for 20 min. Cell lysates were immunoblotted for p-S916-PKD1 and p-S742-PKD1. GAPDH was blotted as a loading control. The experiment was repeated at least three times and representative blots are shown.

To determine whether the compounds are active in cells, we tested their ability to inhibit activation of PKD1 by phorbol 12-myristate 13-acetate (PMA) in LNCaP prostate cancer cells. PKD1 has been shown to be the predominant isoform expressed in these cells [18], and stimulation with PMA leads to PKC-dependent phosphorylation of Ser^738/742 ^in the activation loop followed by autophosphorylation of PKD1 on Ser^916 ^in the C-terminus [8,10]. Since catalytic activity of PKD1 correlates well with the phosphorylation state of Ser^916 ^[10], we measured both p-Ser^916 ^and p-Ser^742 ^levels by Western blot analysis to track PKD1 activity. As is shown in Fig. 4 (*lane 2*), addition of PMA alone induced phosphorylation of both Ser^916 ^and Ser^742 ^of PKD1. When LNCaP cells were pretreated with the novel CID755673 analogs before PMA treatment, concentration-dependent inhibition of phosphorylation at both Ser^916 ^and Ser^742 ^of PKD1 was observed (Fig. 4, *lanes 3-7*). This effect appeared to be most potent for the compound kb-NB142-70, with a calculated cellular IC_50 _for inhibition of Ser^916 ^phosphorylation of 2.2 ± 0.6 μM (n = 3) (Table 2). kb-NB165-09 and kb-NB165-92 showed similar cellular activity, with IC_50_s of 3.1 ± 0.5 (n = 3) and 2.6 ± 0.7 μM (n = 3) respectively. Consistent with our *in vitro *data, kb-NB184-02 was again the least potent compound, demonstrating a cellular IC_50 _of 18.6 ± 2.0 μM (n = 3). GAPDH was used as a loading control instead of PKD1 because the PKD1 antibody showed a slight inconsistency in detecting phosphorylated and non-phosphorylated forms of PKD1 (Fig. 4 and *data not shown*). Taken together, these results indicated that the analogs were capable of inhibiting PKD1 in intact cells.

**Table 2:** Cellular inhibition of PKD1 autophosphorylation at S916 by CID755673 analogs

Compound	Cellular IC_50 _(μM)
kb-NB142-70	2.2 ± 0.6 (n = 3)
kb-NB165-09	3.1 ± 0.5 (n = 3)
kb-NB165-31	8.6 ± 2.0 (n = 3)
kb-NB165-92	2.6 ± 0.7 (n = 2)
kb-NB184-02	18.6 ± 2.0 (n = 3)

Cellular IC_50 _was determined by densitometry analysis of Western blotting data for PKD1 autophosphorylation at S916 in LNCaP cells. Each IC_50 _was calculated as the mean ± S.E.M. of at least two independent experiments. *n, number of independent experiments

### Specificity of CID755673 and its analogs to PKD

**Figure 5:**
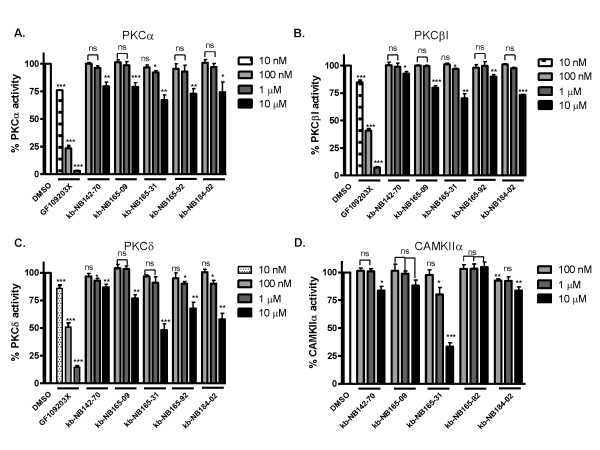
**Selectivity of the CID755673 analogs**. Inhibition of PKCα (***A***), PKCβI (***B***), PKCδ (***C***), or CAMKIIα (***D***) by each of the 5 analogs was determined at 100 nM, 1 μM, and 10 μM concentrations. In the PKC assays, the potent PKC inhibitor GF109203X was used as a control. Data are the mean ± S.E.M. of three independent experiments. Statistical significance was determined using the unpaired t-test. ns, not statistically significant; *, *p *< 0.05; **, *p *< 0.01; ***, *p *< 0.001.

We previously reported that CID755673 showed selectivity toward PKD and did not inhibit several other kinases tested, including PLK1, CAK, protein kinase B (AKT/PKB), PKCα, -βI, -δ, or CAMKIIα. To determine whether the novel analogs retained this specificity, we tested the compounds against their ability to inhibit PKCα, -βI, -δ, and CAMKIIα in *in vitro *radiometric kinase activity assays. All analogs were poor inhibitors of PKCα and PKCβI, with only slight (< 50%) inhibitory activity at 10 μM concentration (Fig. 5A and 5B). This was also true for PKCδ and CAMKIIα with the exception of kb-NB165-31, which did show nearly 50% inhibitory activity toward PKCδ and about 70% inhibition of CAMKIIα activity at 10 μM concentration (Fig. 5C and 5D). As a positive control, the potent PKC inhibitor GF109203X showed strong inhibition of all three of these isoforms (Fig. 5A-C).

To further investigate the specificity of this series of compounds, a kinase profiling experiment was conducted on CID755673, testing 48 additional kinases (Table 3). CID755673 showed "significant" inhibition (≥ 50%) of six out of a total 48 kinases - MK2, GSK-3β, CK1δ, MK5/PRAK, CDK2, and ERK1. As a control, PKD2 activity was reduced by 95% when treated with 10 μM CID755673. A separate, smaller scale analysis of the kinase inhibition profile of the CID755673 analogs has also been conducted and showed similar patterns of inhibition as the parental compound, indicating that the analogs of CID75573 act on similar targets (data not shown).

**Table 3:** Kinase profiling report for CID755673

	CID755673, 10 μM		CID755673, 10 μM
Kinase	Average % Inhibition	Kinase	Average % Inhibition
ABL	8	KDR	8
AKT1	3	**MAPKAPK2**	**95**
AKT2	4	MARK1	12
AMPK	35	MET	18
AurA	7	MSK1	7
BTK	-3	p38a	2
CAMK4	17	p70S6K	44
**CDK2**	**71**	PAK2	3
CHK1	7	PDGFRα	5
CHK2	4	PDK1	22
**CK1δ**	**82**	PIM2	6
c-Raf	2	PKA	6
EGFR	11	PKCη	40
ErbB4	1	PKCγ	30
**Erk1**	**50**	PKCθ	32
Erk2	31	PKCζ	-4
FGFR1	16	**PKD2**	**95**
FLT3	14	PKG1α	13
**GSK3β**	**86**	PKG1β	11
IGF1R	-2	**MK5/PRAK**	**75**
Ikkb	49	RSK1	29
IBSR	3	SGK1	9
IRAK4	0	SRC	7
JNK2	36	SYK	-8

48 kinases were interrogated using a single-dose *in vitro *kinase assay at 10 μM CID755673. -20 to +20% inhibition, baseline levels; >20 to 49% inhibition, compound only marginally actively inhibits the kinase; >50% inhibition, compound is actively inhibiting the kinase. The assay was performed by Caliper Life Sciences (Hanover, MD).

### Effects of the CID755673 analogs on tumor cell death, proliferation, and cell cycle distribution

**Figure 6:**
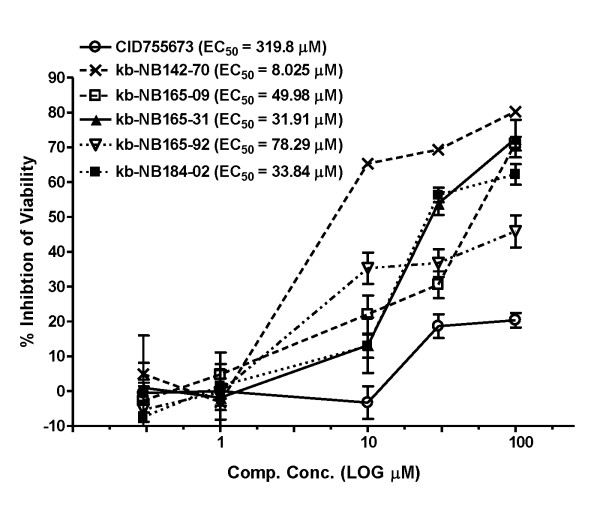
**Cytotoxic effects of the CID755673 analogs in PC3 cells**. PC3 cells were seeded into 96-well plates (3000 cells/well) and were then incubated in media containing 0.3-100 μM inhibitors for 72 h. MTT solution was added to each well and incubated for 4 h. Optical density was read at 570 nm to determine cell viability. The EC_50 _was determined as the mean ± S.E.M. of three independent experiments for each compound.

Given the effects of PKD3 knockdown by siRNA or CID755673 in the inhibition of prostate cancer cell proliferation [18,20] and the implications that PKD regulates cell survival and proliferation [12,29], we wanted to test whether the new compounds were cytotoxic and whether they also inhibited prostate cancer cell proliferation. Therefore, we determined the cytotoxic effects of the compounds on PC3 cells by MTT assay. As shown in Fig. 6, the parental compound induced very little cell death, having an EC_50 _of 319.8 μM in this context. In contrast, the analogs showed considerable increases in cytotoxicity. kb-NB142-70 was again the most potent, causing considerable cell death and demonstrating an EC_50 _of 8.025 μM. kb-NB165-09, kb-NB165-31, and kb-NB184-02 showed similar effects on cell death, with EC_50_s of 49.98 μM, 31.91 μM, and 33.84 μM, respectively.

**Figure 7:**
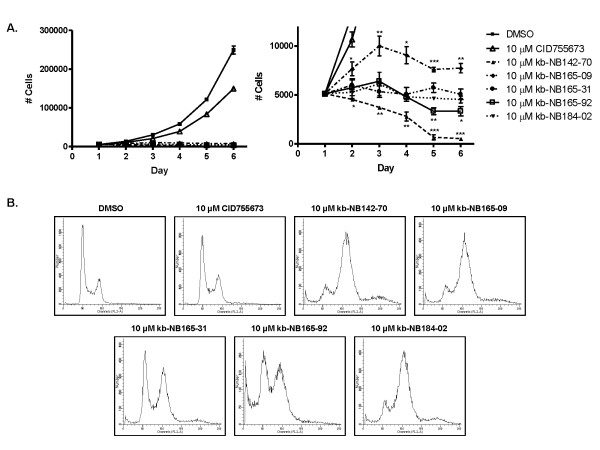
**Effects of the CID755673 analogs on cell proliferation in PC3 cells**. ***A***, The analogs caused potent arrest in cell proliferation. PC3 cells were plated in triplicate in 24-well plates. Cells were allowed to attach overnight. A cell count at day 1 was made, and then either vehicle (DMSO) or the indicated compound at 10 μM concentration was added. Cells were counted daily for a total of 6 days. Media and inhibitor were refreshed every 2 days. The mean cell number ± S.E. was plotted over time. The experiment was repeated twice and a representative graph is shown. Statistical significance versus Day 1 cell count was determined by unpaired t-test and is indicated. *, *p *< 0.05; **, *p *< 0.01; ***, *p *< 0.001. ***B***, The analogs caused G_2_/M phase cell cycle arrest. PC3 cells were treated with either vehicle (DMSO), or 10 μM concentration of indicated compound for 48 hours. Cell cycle distribution was determined by flow cytometry after propidium iodide labeling of fixed cells. The experiment was repeated three times and a representative is shown for each compound.

In addition to the novel analogs demonstrating increased cytotoxicity when compared to the parental compound, they also caused dramatic arrest in prostate cancer cell proliferation when applied at 10 μM concentration to PC3 cells, as determined by cell counts over six consecutive days (Fig. 7A). In contrast to the parental compound, which only slowed cell proliferation, the novel analogs drastically inhibited cell proliferation, with kb-NB142-70 being most potent among the compounds.

To gain insight into the mechanism of growth inhibition caused by the analogs, we conducted cell cycle analysis in PC3 cells. Our previous data indicated the parent compound CID755673 caused G_2_/M phase cell cycle arrest when applied at 10 or 25 μM for six days [20]. In the present study, PC3 cells were treated with 10 μM compound for 48 h and cell cycle distribution was analyzed by flow cytometry after propidium iodide labeling of fixed cells. Indeed, the compounds showed increased accumulation in the G_2_/M phase of the cell cycle when compared to the DMSO treated control or to CID755673 (note that in this experiment, 48 h incubation of CID755673 was too short to induce G_2_/M arrest) (Fig. 7B). Taken together, our data indicated that the novel analogs of CID755673 were potent inhibitors of survival and proliferation in prostate cancer cells.

### CID755673 and its analogs cause accumulation of cyclin D1 and cyclin D3

**Figure 8:**
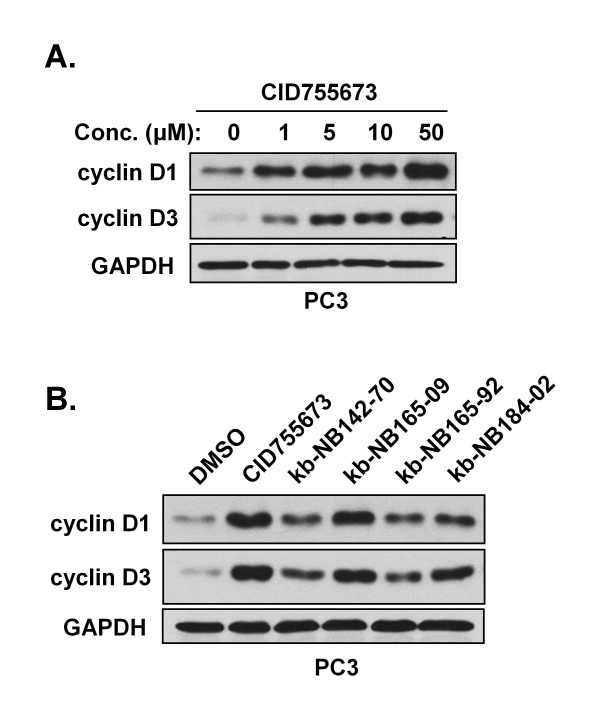
**CID755673 and its analogs cause accumulation of cyclin D1 and cyclin D3**. ***A***, PC3 cells were treated with increasing concentrations of CID755673 for 48 hrs. Inhibitor and growth media were refreshed after 24 hrs. Western blots for cyclin D1 and cyclin D3 are shown. ***B***, PC3 cells were treated with 25 μM CID755673, 10 μM kb-NB142-70, 10 μM kb-NB165-09, 1 μM kb-NB165-92, or 10 μM kb-NB184-02 for 48 hrs. Note that 1 μM kb-NB165-92 was used in this assay since this compound at 10 μM caused significant cell death. Inhibitors and growth media were refreshed after 24 hrs. Western blots for cyclin D1 and D3 are shown. GAPDH was used as a loading control.

Though our evidence supports that CID755673 and its analogs induce cell cycle arrest at G_2_/M phase, a recent study by Torres-Marquez *et al*. demonstrated that CID755673 treatment enhanced phorbol ester- and growth factor-induced DNA synthesis and G_1_/S cell cycle progression in Swiss 3T3 cells independent of PKD1 [30]. In this study, it is important to note that both DNA synthesis and cell cycle distribution were determined after 40 h CID755673 treatment, while in our previous study cell proliferation was measured by counting cell numbers for six consecutive days of CID755673 treatment [20]. Although it was clear based on counting cell numbers that CID755673 inhibited cell proliferation and ultimately caused G_2_/M arrest, our study did not rule out the possibility that this compound could affect other stages of cell cycle progression. To investigate this possibility and to determine if CID755673 indeed affects the G_1_/S transition, we measured the levels of cell cycle markers in response to treatment with CID755673 and its analogs. As shown in Fig. 8A, CID755673 induced cyclin D1 and D3 expression in a concentration-dependent manner in PC3 cells, suggesting a role for CID755673 in promoting the G_1_/S transition. Importantly however, the analogs of CID755673, with the exception of kb-NB165-09, showed much reduced effects on levels of cyclin D1 or D3, implying the specificity of these compounds was improved (Fig. 8B). These data support the idea that CID755673 and its analogs have a complex effect on cell cycle progression; in addition to the induction of G_2_/M arrest and subsequent inhibition of cell proliferation, these compounds may also promote the G_1_/S transition.

### Effects of the CID755673 analogs on tumor cell migration and invasion

**Figure 9:**
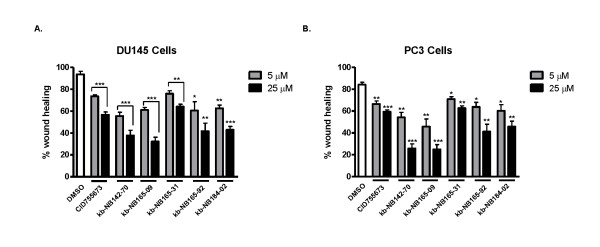
**Effects of the CID755673 analogs on prostate cancer cell migration**. The analogs inhibited wound healing in prostate cancer cells. DU145 cells (***A***) or PC3 cells (***B***) were grown to confluence in 6-well plates. The monolayer was wounded and imaged immediately. Cells were then treated with either vehicle (DMSO) or analogs at indicated concentration for 24 hours. Cells were then fixed and stained with 0.5% crystal violet. Percentage wound closure was calculated as an average of 9 determinations for each concentration/compound as described under "Methods." Data shown are the mean ± S.E.M. for three independent experiments. Statistical significance versus the DMSO control was determined by unpaired t-test in GraphPad Prism V. ns, not statistically significant; *, *p *< 0.05; **, *p *< 0.01; ***, *p *< 0.001.

**Figure 10:**
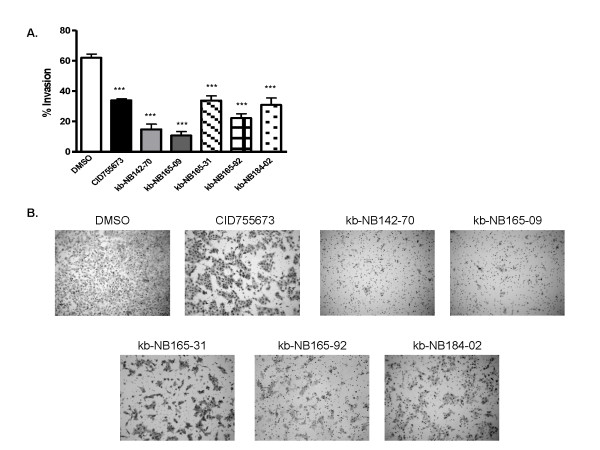
**Analogs of CID755673 inhibit prostate cancer cell invasion**. ***A***, The analogs inhibited invasion in DU145 cells. 0.08 M DU145 cells in RPMI 1640 media containing 0.1% FBS and 10 μM of indicated compound were seeded into Matrigel inserts. After 22 hours, noninvasive cells were removed and invasive cells were fixed in 100% methanol, stained in 0.1% crystal violet solution, and photographed. The number of cells that invaded the Matrigel matrix was determined by cell counts in 5 fields relative to the number of cells that migrated through the control insert. The data shown is the mean ± S.E.M. of two independent experiments. Statistical significance versus the control DMSO was determined by unpaired t-test. ***, *p *< 0.001. ***B***, Representative images comparing invasion of the vehicle (DMSO) and the compounds.

Previous reports have indicated that PKD may have important roles in the regulation of cell motility, adhesion, and invasion [31-33]. Additionally, we previously demonstrated that the PKD inhibitor CID755673 slowed cell migration and invasion in prostate cancer cells [20]. In order to assess whether the novel analogs of CID755673 retained the ability to slow prostate cancer cell migration and invasion, we performed two assays. First, we evaluated the effects of the compounds on migration in both DU145 and PC3 cells by wound healing assay. Confluent cells were wounded and then treated with either 5 μM or 25 μM inhibitor. Wound closure was inhibited in a concentration-dependent manner in both DU145 and PC3 cells (Fig. 9A and 9B). In this assay, kb-NB142-70 and kb-NB165-09 were the most potent inhibitors of wound healing, with wounds showing only 25-35% closure when treated with 25 μM concentration of these two compounds. kb-NB165-31 appeared to strongly resemble the potency of the parental compound, demonstrating 55-60% wound closure at 25 μM concentration in both PC3 and DU145 cells. The analogs also significantly inhibited tumor cell invasion measured by Matrigel invasion assay (Fig. 10A and 10B). Consistent with our previously reported results, 10 μM CID755673 significantly inhibited invasion of DU145 cells. Invasion was also inhibited by kb-NB165-31, kb-NB165-92, and kb-NB184-02 at levels similar to the parental compound. However, kb-NB142-70 and kb-NB165-09 showed increased potency in this assay, reducing percent invasion to only 10%. Taken together, these results support the conclusion that the novel analogs of CID755673 are potent inhibitors of prostate cancer cell migration and invasion.

## Discussion

In this study, we report the generation and characterization of five novel analogs of the PKD inhibitor CID755673. This compound, previously identified as a novel PKD inhibitor, inhibited PKD1 with an IC_50 _of 182 nM *in vitro*, and blocked cancer-associated properties of prostate cancer cells. The novel analogs, synthesized to have modifications in both the core structure and side chains, showed equal or increased potency to PKD1 inhibition *in vitro *and in cells when compared with CID755673. Additionally, we confirmed they also inhibited PKD2 and PKD3 *in vitro*, acting as pan-PKD inhibitors like the parental compound. Of the compounds reported here, the most potent was kb-NB142-70, which inhibited PKD1 with nearly a 7-fold greater potency compared to the parental compound. Furthermore, kb-NB142-70 inhibited PKD2 and PKD3 about 4-fold stronger than CID755673. The analogs also demonstrated increased inhibition of PMA-induced autophosphorylation of endogenous PKD1 in LNCaP prostate cancer cells when compared to the parental compound. Thus, we have established that these small molecule analogs of CID755673 are also potent inhibitors of PKD both *in vitro *and in cells.

CID755673 is superior in specificity when compared with other compounds known to inhibit PKD, such as staurosporine and staurosporine-related the compounds K252a and Gö6976, even though these compounds have been reported to inhibit PKD in the low double- and single-digit nanomolar range (IC_50_s of 40 nM, 7 nM, and 20 nM, respectively). A kinase profiling report demonstrated that CID755673 may also target a few additional kinases, including glycogen synthase kinase-3β (GSK3β), casein kinase 1δ (CK1δ), mitogen-activated protein kinase-activated protein kinase (MK) 5, MK2, and cyclin-dependent kinase 2 (CDK2). Importantly however, CID755673 lacks or shows only marginal activity towards almost all PKC isoforms that have been tested thus far (including PKC-α, -β, -γ, -δ, -η, -θ and -ζ), which distinguishes it from the commonly used PKC/PKD inhibitors such as Gö6976. This feature may allow selective targeting of PKD-mediated signaling pathways and cellular processes, though discretion must be used since additional targets of CID755673 do indeed exist. Similar to the parental compound, the novel analogs for the most part retained specificity when tested against PKCα, -βI, -δ, and CAMKIIα. One compound, kb-NB165-31, did show significant inhibitory activity toward PKCδ and CAMKIIα when tested at 10 μM concentration. This compound has an iodine atom added as a side chain of the benzene ring in kb-NB142-70, which retained strong selectivity to PKD *in vitro*, suggesting that the increase in lipophilicity and the introduction of a polarizable group at the phenol *ortho*-position reduce compound specificity. Interestingly, in the case of kb-NB165-92, the expansion of the lactam by one carbon to a fused 8-membered ring reduced the potencies for PKD1 and PKD2 by 2-4 fold, while not altering potency for PKD3, implying that zone III of our pharmacophore may contain determinants for isoform-selectivity. However, this concept should be further exploited as methoxy analogs of kb-NB142-70 and kb-NB165-92, in contrast, did not exhibit an analogous shift in isoform-selectivity.

Cellular activity of the analogs was demonstrated through inhibition of PMA-induced activation of endogenous PKD1 by measuring the phosphorylation levels of Ser^916 ^and Ser^742^. Based on the canonical pathway of PKC-dependent PKD activation, phorbol ester-stimulated phosphorylation on Ser^738/742 ^by PKC followed by autophosphorylation of PKD1 on Ser^916 ^would result in full activation of PKD [8,10,34,35]. However, recent studies suggest that Ser^742 ^may be a site of both trans- and autophosphorylation. While initial, early catalytic activation of PKD requires rapid transphosphorylation on Ser^738/742 ^by PKC isoenzymes, the major mechanism required to maintain prolonged PKD activation is Ser^742 ^autophosphorylation [11]. Therefore, the observed dose-dependent inhibition of Ser^742 ^phosphorylation on PKD1 after agonist stimulation (100 nM PMA for 20 min) by our novel analogs reflects the inhibition of PKD1 autophosphorylation at this site, analogous to the inhibition of Ser^916 ^phosphorylation. Further analysis is required to determine the precise mechanism of inhibition of PKD by these novel compounds.

PKD has been implicated in the regulation of cell proliferation, survival, and apoptotic pathways in multiple cell types [16,18,36]. We have previously shown that PC3 cells predominantly express high levels of PKD3, potentially making them very sensitive to PKD3 inhibition, and that knockdown of PKD3 by siRNA causes strong arrest in cell proliferation in these cells [18]. Here, we have shown that one of the more striking differences between the parental compound and its analogs is the increase in cytotoxicity and dramatic arrest in cell proliferation. While CID755673 is only minimally cytotoxic to prostate cancer cells, and can be tolerated at high concentrations for prolonged treatments [20], the novel analogs induced significant cytotoxicity in PC3 cells after much shorter treatments (48 h) and at much lower concentrations (5-10 μM). Based on our preliminary analysis, the effects of the compounds on viability in other prostate cancer cells (LNCaP and DU145) are comparable to those in PC3 cells (data not shown). The inhibitors appear to exhibit a general inhibitory effect on cell viability, with potency varying between different tumor cell types. Additionally, the analogs cause much more potent arrest in cell proliferation than the parental compound. Since the anti-proliferative effects of the analogs phenocopied those caused by knockdown of PKD3 in PC3 cells, it is conceivable that these effects, at least to some extent, are mediated through inhibition of PKD. That said, we cannot exclude the possibility that CID755673 and its analogs have additional cellular targets whose inhibition may contribute to the elevated cytotoxicity and potent growth arrest observed in prostate cancer cells. Moreover, since the analogs, mimicking the parental compound, all induced apparent G_2_/M cell cycle arrest, it is likely that the mechanisms underlying the growth inhibition caused by the analogs are similar to those induced by the parental compound. Based on the kinase profiling data, we speculate that, in addition to PKD, the inhibitory effect of CID755673 and its analogs on cell proliferation may be contributed to the inhibition of CDK2, another potential target of CID755673. Although CDK2 is generally considered a regulator of S-phase entry [37,38], some reports have also linked it to the G_2_/M transition [38,39]. According to the accepted model of cell cycle progression, CDK2 is activated by binding to cyclin E in late G_1 _phase, resulting in phosphorylation of the retinoblastoma protein (Rb) and facilitating the G_1_/S-phase transition [40]. It also promotes progression of S-phase by binding to cyclin A. However, it has been reported that inhibition of CDK2 by expression of a dominant negative CDK2 mutant or overexpression of p27^kip1 ^can cause accumulation in G_2_/M [38,39]. Therefore, it is plausible that the G_2_/M arrest and reduced cell proliferation caused by CID755673 and its analogs is in part due to inhibition of CDK2. It is also possible that CID755673 and its analogs may inhibit other members of the CDK family, for example CDK1, which plays a critical role in G_2_/M cell cycle progression. Finally, it must be stated that although CKD2 and a few other proteins were identified as potential hits in a single dose kinase profiling experiment, the activities of CID755673 and its analogs toward these targets need to be further validated in 10-point dose-response kinase assays.

Although CID755673 and its analogs potently inhibited cell proliferation, their effects on cell cycle progression appeared to complex, involving two opposing effects on different stages of the cell cycle: 1) promotion of the G_1_/S transition; 2) induction of G_2_/M arrest. The G_2_/M arrest ultimately leads to cessation of cell proliferation. Our findings that CID755673 and its analogs induced cyclin D1 and D3 expression may underlie the potentiation effect of CID755673 on the G_1_/S transition induced by other mitogens [30]. Given that the report by Torres-Marquez *et al*. used DNA synthesis and cell cycle distribution as readouts, it remains to be determined if the potentiation effect reported indeed resulted in increased cell number (cell proliferation) since the G_2_/M block may ultimately inhibit this effect. With regard to the potential targets that may account for this effect, we hypothesize, based on our kinase profiling data, that GSK-3β could play a role since active GSK-3β has a negative effect on cell cycle progression [41]. Expression of the cell cycle proteins cyclin D1 and cyclin D3 is regulated by GSK-3β signaling at the transcriptional level and through protein degradation [41-43]. Thus, inhibition of GSK-3β may be in part responsible for the promotion of the G_1_/S transition and the reported potentiation effect with other mitogens. It is important to note that the analogs of CID755673 in general showed less activity in inducing cyclin D1 or D3 expression, suggesting that they are less active at promoting the G_1_/S transition and are more selective for PKD. This correlated to their much enhanced growth suppressive and cytotoxic effects in prostate cancer cells, implying that reducing/removing the G_1_/S cell cycle-promoting effect of the analogs could significantly improve the antitumor activity of these analogs.

In addition to the effects of these analogs on cell survival and proliferation, we also show that they are potent inhibitors of prostate cancer cell migration and invasion. kb-NB142-70 and kb-NB165-09 in particular, strongly reduced wound healing in both DU145 cells and PC3 cells in a dose-dependent manner, and significantly inhibited invasion of DU145 cells through Matrigel invasion inserts when applied at 10 μM concentration. Furthermore, the pattern of inhibition exhibited by the analogs is fairly consistent with their inhibitory activities toward PKD. This suggests an important role for PKD in prostate cancer cell motility and supports the potential value of therapeutic targeting of PKD in the reduction or prevention of prostate tumor metastases. Though the mechanism through which PKD may mediate migration and invasion is not yet known, several recent reports have begun to shed light onto the complexity of these signaling pathways, suggesting PKD involvement in both β-catenin and Akt signaling in prostate cancer cells [18,19,32].

## Conclusions

In conclusion, we report the biochemical and functional analysis of several novel analogs of the PKD inhibitor CID755673. These analogs show equal and increased potency toward PKD inhibition both *in vitro *and in cells. The new lead compounds display prominent cytotoxic and anti-proliferative effects, and potently inhibit migration and invasion in prostate cancer cells. Although the molecular mechanisms underlying some of the biological effects of these compounds appear to be complex and may involve additional targets, their potent effects on multiple cancer-associated biologies warrant further development of this series of compounds toward possible clinical application in cancer therapy.

## Abbreviations

PKD: protein kinase D; CID755673: 7-hydroxy-2,3,4,5-tetrahydro-[1]benzoxolo[2,3-c]azepin-1-one; PKC: protein kinase C; DAG: diacylglycerol; PMA: phorbol 12-myristate 13-acetate; VEGF: vascular endothelial growth factor; ERK1/2: extracellular signal-regulated kinase 1/2; HDAC: histone deacetylase; AKT/PKB: protein kinase B; GSK-3β: glycogen synthase kinase-3β; CK1δ: casein kinase 1δ; MK: mitogen-activated protein kinase-activated protein kinase; CDK: cyclin-dependent kinase; DMSO: dimethyl sulfoxide; PH: pleckstrin homology; CAMK: calcium/calmodulin-dependent kinase; MTT: 3-(4,5-Dimethylthiazol-2-yl)-2,5-diphenyltetrazolium bromide methyl thiazolyl tetrazolium

## Authors' contributions

CRL carried out the proliferation and migration studies, participated in the initial activity screening, *in vitro *activity assays, and cellular activity assays, and drafted the manuscript. KBA carried out the synthesis of the compounds, participated in the design of the compounds, and helped draft the manuscript. KVG carried out the invasion studies and participated in the initial activity screening, *in vitro *activity assays, and cellular activity assays. JC carried out the MTT assay and participated in the initial activity screening and *in vitro *activity assays. ERS participated in the initial activity screening and helped with data interpretation. JSL participated in the design of the study and provided additional guidance. PW participated in the design and synthesis of the compounds, aided in the conception and design the study, and helped draft the manuscript. QWJ conceived of the study, participated in its design and coordination, and helped draft the manuscript. All authors read and approved the final manuscript.
